# Effect of Microstructure on the Dimensional Stability of Extruded Pure Aluminum

**DOI:** 10.3390/ma14174797

**Published:** 2021-08-24

**Authors:** Linlin Fu, Gaohui Wu, Chang Zhou, Ziyang Xiu, Wenshu Yang, Jing Qiao

**Affiliations:** School of Material Science and Engineering, Harbin Institute of Technology, Harbin 150001, China; 19b909032@stu.hit.edu.cn (L.F.); changzhou@hit.edu.cn (C.Z.)

**Keywords:** extruded pure aluminum, texture, annealing, dimensional stability, thermal cycling

## Abstract

High-performance extruded aluminum alloys with complex textures suffer significant dimension variation under environmental temperature fluctuations, dramatically decreasing the precision of navigation systems. This research mainly focuses on the effect of the texture of extruded pure aluminum on its dimensional stability after various annealing processes. The result reveals that a significant increment in the area fraction of recrystallized grains with <100> orientation and a decrement in the area fraction of grains with <111> orientation were found with increasing annealing temperature. Moreover, with the annealing temperature increasing from 150 °C to 400 °C, the residual plastic strain after twelve thermal cycles with a temperature range of 120 °C was changed from −1.6 × 10^−5^ to −4.5 × 10^−5^. The large amount of equiaxed grains with <100> orientation was formed in the microstructure of the extruded pure aluminum and the average grain size was decreased during thermal cycling. The area fraction of grain with <100> crystallographic orientation of the sample annealed at 400 °C after thermal cycling was 30.9% higher than annealed at 350 °C (23.7%) or at 150 °C (18.7%). It is attributed to the increase in the proportion of recrystallization grains with <100> direction as the annealing temperature increases, provided more nucleation sites for the formation of fine equiaxed grains with <100> orientation. The main orientation of the texture was rotated from parallel to <111> to parallel to <100> after thermal cycling. The change in the orientation of grains contributed to a change in interplanar spacing, which explains the change in the dimension along the extrusion direction during thermal cycling.

## 1. Introduction

Aluminum alloys are widely considered candidate structural materials in the inertial navigation sector because of their high strength-to-weight ratio and low density [1,2]. However, dimensional change in aluminum alloys arises spontaneously during service, which is insufficient to meet the accuracy requirements of inertial navigation devices [3]. The irreversible strain of the structural materials beyond 10^−6^~10^−7^ would bring about the navigation error of the aerospace instruments, resulting in satellite deviation and major aviation disaster [3,4,5].

The influencing factors of dimensional stability are complicated and include multiple factors such as residual stress, precipitated phase, microstructure, etc. Previous research on the dimensional stability of alloys was limited to the establishment of the relationship between the macroscopic process and dimensional stability. Uju et al. [1] found that residual plastic strain of the Al–Mg alloy induced by thermal cycling was decreased with the addition of fly ash. Song et al. [6] reported a method to improve the dimensional stability of an Al–Mg–Cu alloy by stress aging. The increase of the applied external stress contributed to the decrease of the internal stress and a decrease of the residual plastic strain. Wang et al. [7] investigated the effect of heat-treatment on the dimensional stability of 3D-printed continuous carbon-fiber-reinforced composites and concluded that the best heat-treatment condition is 100 °C for 8 h, which would obtain better stability and mechanical properties for the 3D printed continuous carbon fiber reinforced composites. Research on the impact of a targeted single variable on dimensional stability and high-precision analysis is urgently needed in order to obtain the dimensional stability mechanism. Moreover, the temperature disturbance caused by the cycle of the four seasons or the alternation of day and night is inevitable for the material. The thermal cycling is the prime environmental factor for the dimensional stability of materials [8,9,10].

Extrusion and rolling are common deformation processes to improve the strength and toughness of aluminum alloys [11,12]. Jandaghi et al. [13] researched rolling as a supplementary deformation process that could accelerate the microstructural refinement of the Al–Mn–Si specimens. Moreover, the direct-rolling of samples after constrained groove pressing was more susceptible for strength enhancement compared to cross-rolling. The optimum achieved values for yield and ultimate tensile strength were 155 and 197 MPa, respectively. Pouraliakbar et al. [14] used a genetic programming approach to predict the effect of a constrained groove pressing on the grain size of aluminium and reported that the final grain size of constrained-groove pressed aluminum sheets could be precisely predictable by both the mechanical and physical properties of specimens, which saves the time and expenditure of the experimental process. The texture structure after deformation is an important material characteristic that plays a key role in controlling the thermophysical, mechanical properties and dimensional stability of the material [11,15]. The residual plastic strain along the axial direction of the extruded LY12 Al alloy after thermal cycling was about 13 times than that along the transverse direction [15]. The rolled alloy sheet investigated by several scholars demonstrates significant anisotropy in micro-yield, creep deformation and tensile properties due to the strong texture [16,17,18,19]. The strength degradation of commercially pure Al after annealing was the result of the evolution of texture and sub-grain growth during the recovery and recrystallization, which depended on the annealing temperature [20]. However, the current research has not established a clear relationship between the evolution of texture and dimensional changes under temperature disturbance [21].

The common methods for adjusting the texture component of an aluminum alloy include: annealing, adjusting the alloy composition and multiple deformations, etc. [22,23]. In recent years, annealing heat treatment has attracted wide attention due to its advantages of easy operation, mature technology and low cost [24,25,26]. For the most pure Al alloys, the recovery process starts at 95 °C~150 °C, while the recrystallization process starts at 200 °C~300 °C [27]. A temperature around 150 °C is required for the residual internal stress relief. The texture components of aluminum alloy after deformation are mainly composed of Cube {001} <100>, Copper {112} <111>, S {123} <634> and Brass {110} <112>. An increment in the area fraction of grains with <100> orientation and a decrement in the area fraction of grains with <111> orientation were found in the extruded pure aluminum annealed at above the recrystallization temperature by J.P. Hou, indicating the transformation of texture from the <111> to the <100> [20]. The strong Brass {110} <112>, Copper {111} <112>, S {123} <634> and R {124} <112> components and some weak S/Brass {414} <234> and Goss {110} <001> were found in the rolled aluminum alloy sheet after annealing [28]. The texture composition is controlled by the annealing temperature and the deformation process [29,30].

In this study, extruded pure aluminum was annealed at 150 °C/1 h, 350 °C/1 h and 400 °C/1 h to prepare microstructures with diverse texture compositions, to definite the effect of texture on the dimensional stability of aluminum alloy and explore the kinetics of texture evolution under thermal cycling. Pure aluminum was chosen to remove effects caused by alloy elements and precipitated phase. The dimensional stability of pure aluminum with various textures was characterized by thermal cycling. The obvious grain refinement and change of the orientation of grains caused by thermal cycling were observed by the electron backscatter diffraction technique. The relationship between the change of crystal orientation and the residual plastic strain was established.

## 2. Materials and Methods

**Figure 1:**
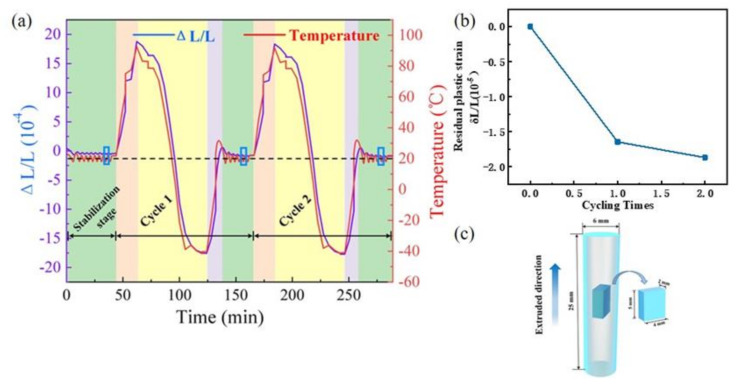
(**a**) The curves of the temperature and thermal strain along the extrusion direction during the thermal cycling test. (**b**) The curve of the residual plastic strain, which is calculated from the strain marked by the blue box in (**a**) and the number of thermal cycles. (**c**) Schematic diagram of the sampling direction of the EBSD experiment.

In this study, the raw material was Φ 36 × 50 mm pure aluminum bar (99.99%, Northeast Light Alloy Co., Ltd., Harbin, China). It was cut into a series of Φ 6 × 25 mm standard round bar samples with the axis parallel to the extrusion direction by electrical discharge machining. The samples were annealed at 150 °C/1 h, 350 °C/1 h and 400 °C/1 h to release residual stress and obtain various texture structures [31,32]. The texture structures were examined by electron backscatter diffraction (EBSD) using a FEI Quanta 3D FEGSEM instrument (FEI Company, Hillsboro, OR, USA). The samples for EBSD investigation were mechanically cut into slices with a size of 5 mm × 4 mm × 2 mm in the longitudinal section as shown in Figure 1c and polished in an HClO_4_: C_2_H_5_COOH solution at −35 °C for 60 s.

In order to evaluate the dimensional stability of pure aluminum with various textures, the residual plastic strain was tested by a thermal cycling experiment using the NETSCH DIL 402 C instrument. The thermal cycling experiment was widely used to detect the dimensional stability of metal due to its high accuracy and short detection cycle [33,34]. The standard round bar samples obtained by the electrical discharge machining were used for the thermal cycling experiment. The DIL 402 C instrument dual mandrel dilatometer (NETSCH Co. Ltd., Selb, Germany) with a high-purity quartz sample holder and an ejector with a low thermal expansion coefficient and ±1% error; the displacement sensor is made of Invar with a resolution of 0.125 nm [3]. Displacement sensors were distributed at both ends of the round bar sample to detect the size change in the length direction of the sample. The measurements were performed under a nitrogen gas atmosphere to avoid the oxidation of the samples. The dimensional change of extruded pure aluminum, as well as the change of temperature, was the real-time output during the thermal cycling process. Figure 1a shows the variation of temperature and thermal strain along the extrusion direction with time during the thermal cycling test.

According to the application temperature range of aluminum alloy in the field of precision instruments [33,35,36], the samples were cycled between −40 °C and 80 °C. Dong [33] and W.A. Uju [1] et al. found that the change of size after ten thermal cycles could clearly reflect the dimensional stability of the material. In this study, the number of times in thermal cycles was increased to 12 based on the detection capability of the instrument. Thus, the sample was cycled for twelve cycles between high temperature (80 °C) and low temperature (−40 °C) with a heating and cooling rate of 5 °C/min. The evolution of dimensions in the length direction were concerned. The residual plastic strain, which represented the relative strain along the length of the specimen formed after thermal cycling, was tested to characterize the dimensional stability during the thermal cycling process. After each cycle, the sample was stabilized at 20 °C for half an hour to ensure the equilibrium of temperature and calculate the residual plastic strain as shown in Figure 1b. The larger the value of residual plastic strain, the worse the dimensional stability of the material.

## 3. Results and Discussion

### 3.1. EBSD Analysis of Extruded Al after Annealing

**Figure 2:**
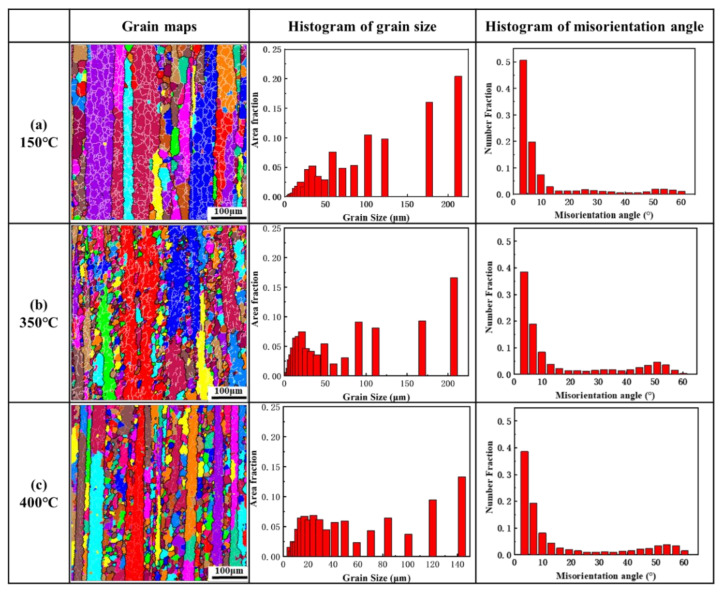
(**a**) Grain structures, histograms of grain size and histograms of the misorientation angle of the samples annealed at (**a**) 150 °C, (**b**) 350 °C and(**c**) 400 °C before thermal cycling. Dark lines correspond to HAGBs and white to LAGBs.

The microstructures of the as-deformed pure aluminum samples after annealing heat treatments were investigated by electron backscatter diffraction (EBSD). The EBSD images, the histograms of grain size and the histograms of the misorientation angle of the samples in the longitudinal section after annealing at 150 °C, 350 °C and 400 °C are shown in Figure 2. The white lines and black lines indicate the low-angle grain boundaries (LAGBs) and the high-angle grain boundaries (HAGBs). The misorientation angles of the LAGBs and HAGBs were θ ≤ 15°and θ > 15°, respectively. For the specimen of extruded pure aluminum annealed at 150 °C, elongated grains were observed along the extrusion direction and an amount of sub-grains were also observed within the elongated grains, which indicated the occurrence of recovery during the annealing treatment [37]. A significant increment in the amount of HAGBs and a decrement in the amount of LAGBs were found when annealing at 350 °C and 400 °C as shown in Figure 2b, c. Moreover, the area fraction of recrystallized grains annealed at 350 °C and 400 °C were 38.6% and 44.3%, respectively. This phenomenon indicated that the dislocations were absorbed by the boundaries of sub-grains during annealing at 350 °C and 400 °C, increasing the misorientation of LAGBs until they transformed into HAGBs. The recrystallized grain structure and the complex network of grain boundaries were developed when the annealing temperature was 350 °C or 400 °C. The evolution of the microstructure during annealing could be classified into two stages, recovery and nucleation in the range of 150~350 °C, and significant partial recrystallization at above 350 °C. Some researchers had reported that the transformation of texture was complicated in the process of recrystallization [38,39,40].

**Figure 3:**
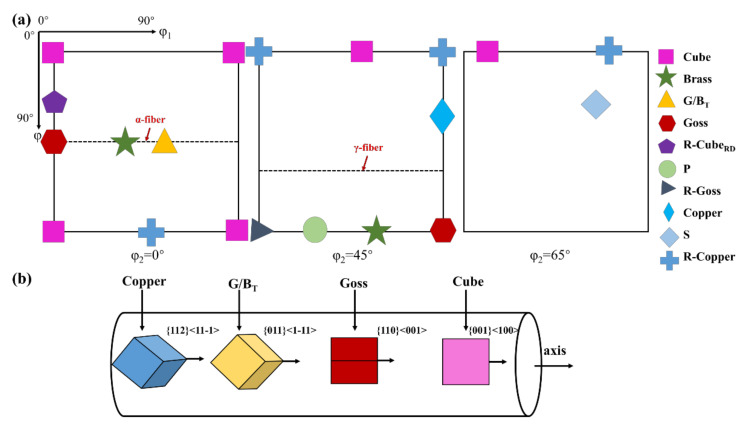
(**a**) ODF sections (at φ_2_ = 0°, 45° and 65°) of the common texture components in aluminum alloy. (**b**) the schematic diagram of the relationship between the orientation of textures: Copper, G/B_T_, Goss and Cube textures and the macroscopic orientation of samples.

**Figure 4:**
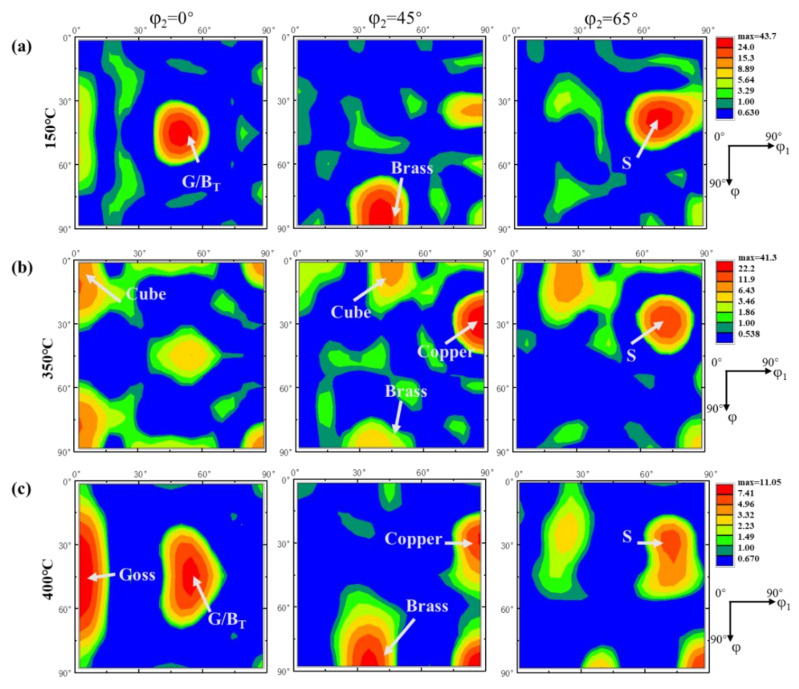
The φ_2_ =0°, 45° and 65° ODF sections of the samples after annealing at (**a**) 150 °C, (**b**) 350 °C and (**c**) 400 °C before thermal cycling.

To better understand the complex texture components of aluminum, Table 1 lists the Euler coordinates and the corresponding Miller index of common textures in aluminum. Figure 3a displays the schematic diagram of the position of the typical texture in ODF section maps of φ_2_ = 0°, 45°, 65°. Figure 4 represents the ODF maps of the texture component of the samples after annealing. The dominating components of the texture of extruded aluminum annealed at 150 °C were composed of the G/B_T_, Brass and S. Annealed at 350 °C, the area fractions of Cube and Copper were increased and the G/B_T_, S and Brass were decreased. Based on previous research [41,42], the evolution of the texture in the Euler space preferentially tends to be conducted through the rotations around the eight <111> axis of slipping octahedral and the <001> and <011> axes of cubic cells. It could be inferred that the G/B_T_ was transformed according to the following approach: G/B_T_ → Copper → Cube → Goss, due to the near 30° <111> relation between Copper and G/B_T_ and the approximate 40° <111> orientation relationship existing between Copper and Cube [22,43]. Moreover, there was also an approximate 40° <111> orientation relationship between the S and Cube, causing the S texture to translate into the Cube texture. Especially, the intensity of texture was significantly weakened at an annealing temperature of 400 °C, wherein the maximum value of intensity (11.1) was only a quarter of that annealed at 350 °C (41.3). This result was consistent with the evolution of the texture of rolled aluminum alloys during the annealing process reported by Shen et al. [22]. It was attributed to the transformation of Copper into Goss texture and the corresponding rebound of the S, Brass and G/B_T_ orientations led to the weakening of the overall texture intensity. In summary, the Goss and Cube textures had a higher nucleation rate at the shear band of the extruded aluminum alloy, resulting in the nucleation of Goss and Cube textures dominating the early stage of recrystallization. With the progress in recrystallization, several new grains with random orientation were formed in the microstructure [44,45]. The schematic diagram of the relationship between the orientation of textures: Copper, G/B_T_, Goss and Cube textures and the macroscopic orientation of samples is shown in Figure 3b. It indicates the evolution of the orientation of grain relative to the macroscopic direction of the sample when the primary textures of the extruded pure aluminum were transformed into each other.

The quantitative analysis of the distribution and area fraction of textures is shown in the following content.

**Figure 5:**
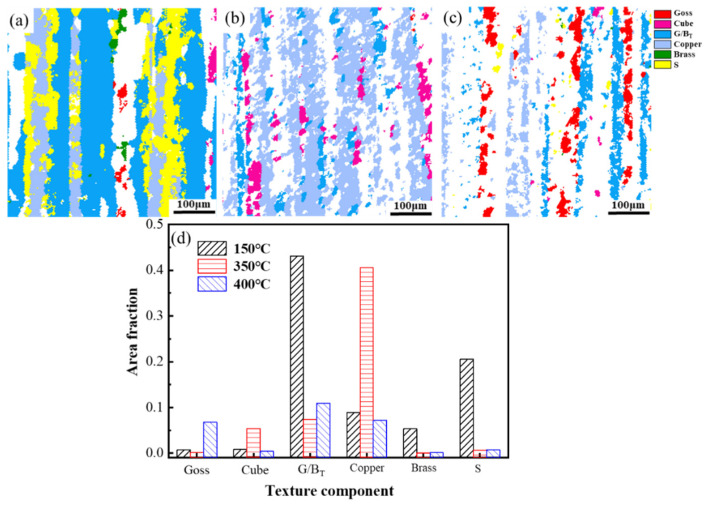
The maps of the main texture in the specimens annealed at (**a**) 150 °C, (**b**) 350 °C and (**c**) 400 °C before thermal cycling. (**d**) Area fraction of main texture components.

The distributions of grain orientations annealed at 150 °C, 350 °C and 400 °C are shown in Figure 5a–c, respectively. Figure 5d demonstrates the area fractions of common textures annealed at 150 °C, 350 °C and 400 °C. The orientations of recrystallized grains were dominated by Goss and Cube textures for all specimens. The orientations of the elongated grains were dominated by Copper and G/B_T_ textures. During the recrystallization process, some newly formed grains with Cube orientation were distributed around the original grains with Cube orientation. Some scientists had reported that the small grains with Cube orientation originated from the deformed Cube substructure [46,47]. The area fraction of <100> texture was increased from 1.5% to 5.5% in the temperature range from 150 °C to 350 °C. Then the increment remained stable until reaching the largest value of 7.2% at 400 °C. On the contrary, the area fraction of <111> texture decreased from 52.0% to 48.1% in the temperature range from 150 °C to 350 °C, then decreased rapidly to 18.1% with the further increase of the annealing temperature.

This reveals that the primary orientation of texture varies from being parallel to <111> to parallel to <100> during the recrystallization process.

### 3.2. Thermal Cycling Dimensional Stability

**Figure 6:**
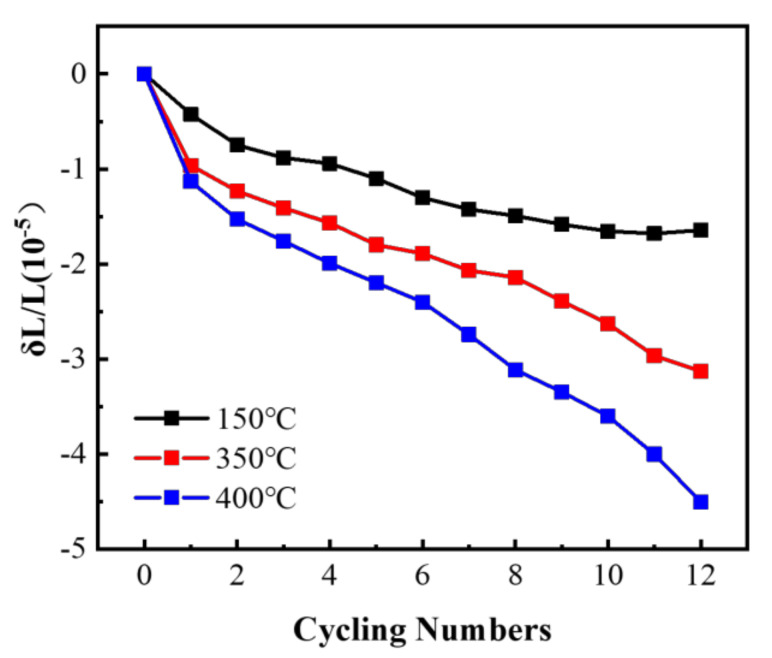
Variation of the residual plastic strain of the sample after annealing with the number of thermal cycles.

Figure 6 shows the relationship between the residual plastic strain of the annealed sample and the number of thermal cycles under the condition of thermal cycling with a temperature range (ΔT) of 120 °C. The dimensional stability was assessed by the value of the residual plastic strain, which had a strong dependence on the number of cycles and textures. The residual plastic strain of the samples was negative, indicating that the macro size decreases under thermal cycling conditions. The residual plastic strain was gradually accumulated by the number of thermal cycling. The residual plastic strain of the sample annealed at 150 °C was increased to 1.58 × 10^−5^ with the number of cycles increased from 1 to 9. Then the increment was inconspicuous until the number of cycles reached 12. The residual plastic strains of the samples annealed at 350 °C and 400 °C caused by single thermal cycle were gradually decreased as the number of thermal cycles increased. The residual plastic strains were reached −1.6 × 10^−5^, −2.2 × 10^−5^ and −4.1 × 10^−5^, with a continuous decline after 12 thermal cycles corresponding to the annealed temperature of 150 °C, 350 °C and 400 °C, respectively. The smaller absolute value of the residual plastic strain indicates that the size of the material is more stable [48]. Thus, the specimens with an annealed temperature of 150 °C displayed a more stable performance than that of 350 °C and 400 °C during thermal cycling. It could be validated that the residual plastic strain of extruded pure aluminum under thermal cycling conditions was increased with the decrement of the area fraction of <111> orientation and the increment of the area fraction of <100> orientation.

The evolution of microstructures, such as the slip of dislocations, the migration of the grain boundaries and the change of the orientation of grains, is the essential reason for the irreversible deformation of materials. Y. Zeng et al. reported that different grain orientations led to different strength mechanisms [49]. The orientation of grains has a significant effect on the rotation of the grain boundary accompanying the texture evolution and the resistance to the movement of dislocations. The competitive relation was observed by Shen et al. between the CSL grain boundary rotation along <111> axis and the other axes [43]. This reveals that the varying texture composition is the main reason for the different dimensional stability of pure aluminum annealed at 150 °C, 350 °C and 400 °C during thermal cycling. Further, the microstructures after thermal cycling were analyzed to explore the structure evolution behavior and mechanism of effect of texture on dimensional stability during thermal cycling, in the next section.

### 3.3. EBSD Analysis of Microstructure after Thermal Cycling

**Figure 7:**
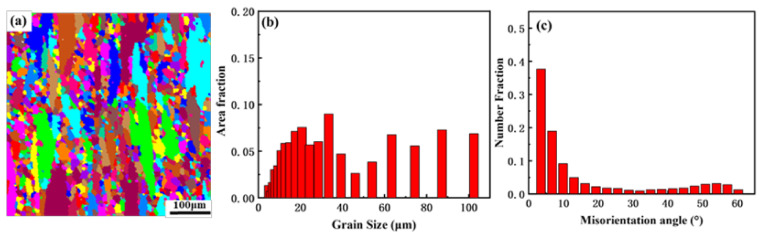
(**a**) Grain structure, (**b**) histogram of grain size and (**c**) histogram of the misorientation angle of the sample annealed at 350 °C after thermal cycling (N = 5).

**Figure 8:**
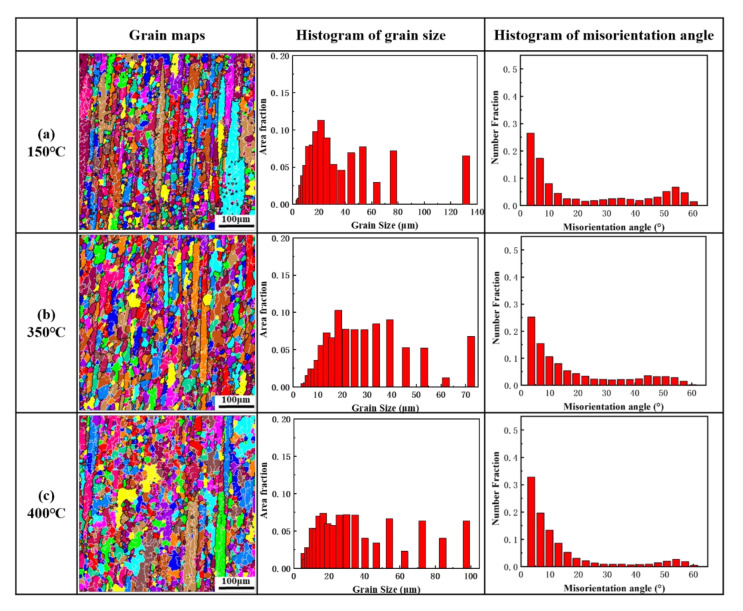
Grain structures, histograms of grain size and histograms of the misorientation angle of samples annealed at (**a**) 150 °C, (**b**) 350 °C and (**c**) 400 °C after thermal cycling (N = 12).

**Figure 9:**
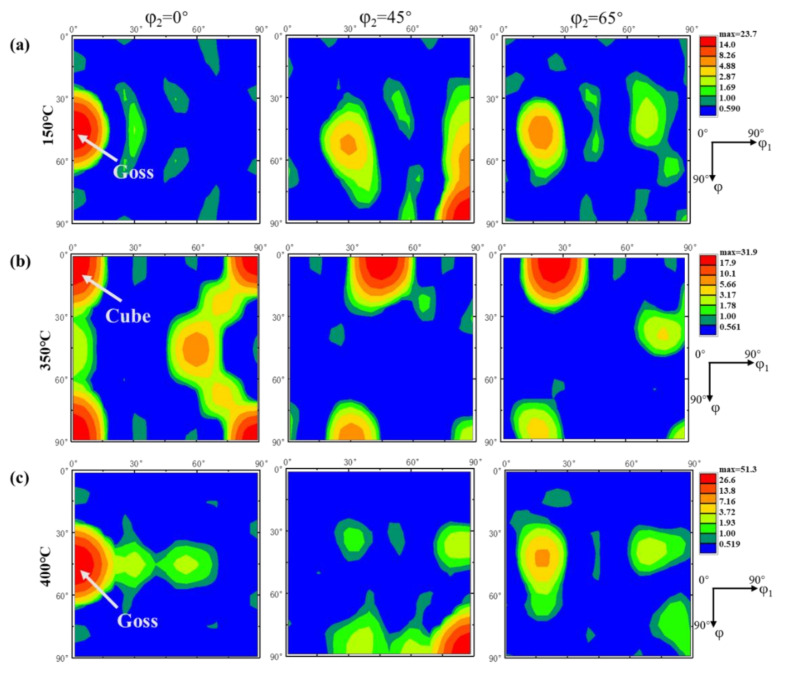
The φ_2_ = 0°, 45° and 65° ODF sections of the samples annealed at (**a**) 150 °C (**b**) 350 °C and (**c**) 400 °C after thermal cycling (N = 12).

The grain structure, histogram of grain size and histogram of the misorientation angle of the sample annealed at 350 °C after five times of thermal cycling are presented in Figure 7. The grain size of the sample annealed at 350 °C after five times of thermal cycling was refined compared to the grain size of the samples before the thermal cycling. The formation of fine grains was attributed to the low-angle grain boundaries absorbing the dislocations to form high-angle grain boundaries. Xie et al. [2,9] reported that the same experimental conditions for each thermal cycle contributed to the same evolution behavior of the microstructure after each cycle. In order to better compare the evolution behavior of microstructure annealed at 150 °C, 350 °C and 400 °C after thermal cycling, the grain structures and the orientation of grains of the samples annealed at 150 °C, 350 °C and 400 °C after twelve times of thermal cycling were characterized as shown in Figure 8 and Figure 9.

Figure 8 shows the grain structure of the samples after twelve thermal cycles with the ΔT of 120 °C. A greater number of equiaxed grains were observed in the original deformed structure. The area fraction of equiaxed grains in samples annealed at 150 °C, 350 °C and 400 °C after thermal cycling were 49.9%, 63.9% and 67.0%, respectively, demonstrating that the area fraction of equiaxed grains was increased with the increase of the annealing temperature. The complex network of LAGBs was developed during thermal cycling, as can be seen from the histograms of the misorientation angle. It could be validated that the complex stress state was induced by thermal cycling, resulting in the formation of a dislocation network and substructures [50,51]. The misorientation of these substructures was gradually increased as the number of thermal cycles increasing until equiaxed grains were formed. Furthermore, the average grain sizes in samples annealed at 150 °C, 350 °C and 400 °C after thermal cycling were reduced from 120.2 μm to 36.3 μm, from 82.4 μm to 28.5 μm and from 58.7 μm to 35.4 μm, respectively, indicating that the increase of the number of equiaxed grains contributed to the decrease of the average grain size and that there was a limit value of about 30 μm in the grain size after the thermal cycling of ΔT = 120 °C.

The change of defects and the change of the orientation of grains that contributed to the dimensional instability may be caused by the formation of the equiaxed grains [15,19]. Thus, the formation of equiaxed grains may be the key factor in the dimensional instability of materials during thermal cycling. The degree of the grain refinement of the sample annealed at 400 °C was lower than that of the sample annealed at 150 °C and 350 °C, while the residual plastic strain was higher after thermal cycling. Thus, the rotation of grains leading to a change in the orientation of the grains parallel to the macroscopic direction is the key to the residual plastic strain of the material during the thermal cycling. The evolution of texture during the formation of equiaxed grains was further analyzed in this study.

Figure 9 shows the ODFs of the samples annealed at 150 °C, 350 °C and 400 °C after thermal cycling. An evident transformation of the texture after thermal cycles was present, typical for the transformation of Copper and G/B_T_ textures into Goss or Cube textures. The main textures of the samples annealed at 150 °C, 350 °C and 400 °C were Goss, Cube and Goss textures, respectively. The <100> orientation dominated the sample after thermal cycling, which was may attributed to the low stored energy and presence of growth-favorable 40° <111> boundaries of the <100> texture [52]. It is worth noting that the intensity of texture annealed at 400 °C after thermal cycling was significantly higher than before. It indicates that the mechanism of the phenomenon that the orientations of the grains were aligned along the <100> direction induced by thermal cycling was different from that in the recrystallization process. The intensity of the texture with a <100> orientation could be continuously increased during the thermal cycling, but that first increased and then decreased during the recrystallization process [11].

Moreover, the texture components after thermal cycling were related to the original texture components by comparing the textures before thermal cycling. It can be inferred that the formation of the <100> texture was dependent on the original grains with the <100> orientation. In the temperature range of 150 °C~400 °C, the increment of the area fraction of grains with <100> orientation contributed to the formation of more equiaxed grains with <100> orientation. The maximum value of the intensity of the texture was increased from 23.7 to 51.3 as the annealing temperature increased.

**Figure 10:**
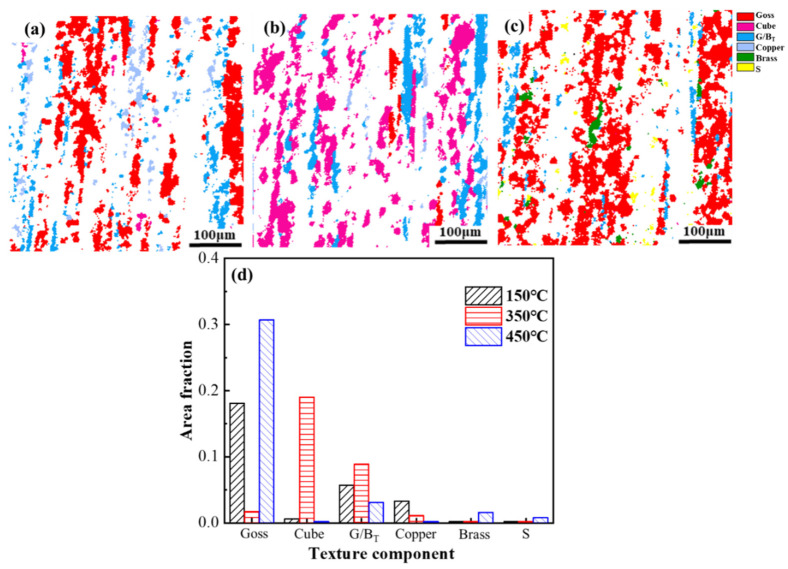
The maps of the main texture in the specimens annealed at (**a**) 150 °C, (**b**) 350 °C and (**c**) 400 °C after thermal cycling. (**d**) Area fraction of main texture components after thermal cycling (N = 12).

In order to characterize the changing of textures mentioned above quantitatively, the area fractions were calculated precisely as shown in Figure 10d. Figure 10a–c show the distribution of textures in the grain structure of the sample annealed at 150 °C, 350 °C and 400 °C after thermal cycling, respectively. Grains with <100> orientation were formed by attaching to the original recrystallized grains, growing along the boundary of the elongated grains [52,53,54]. A clear dividing boundary was formed between equiaxed grains and deformed grains. The area fractions of the grains with <100> orientation of the samples annealed at 150 °C, 350 °C and 400 °C were 18.7%, 23.7% and 30.9%. The interplanar spacing of crystal planes changed with the change of crystallographic orientation [55], such as the <100> crystallographic orientation of aluminum having an interplanar spacing of 2.03 Å, while the <111> orientation has an interplanar spacing of 2.34 Å [56]. The crystallographic orientations along the test direction were rotated from parallel to <111> orientation to parallel to <100> orientation during thermal cycling, leading to a reduction in the macroscopic size. This can explain the results of the thermal cycling test. The rotation of grains leading to a change in the orientation of the grains parallel to the macroscopic direction is the key to the residual plastic strain of the material during thermal cycling.

## 4. Conclusions

In this work, pure aluminum with various texture structures, which were fabricated by annealing heat treatment at 150 °C, 350 °C and 400 °C after deformation, were investigated in dimensional stability and the kinetics of texture evolution. The main results of the current study are summarized as follows:

1. An annealing temperature of 150 °C led to extruded pure aluminum with the best dimensional stability during thermal cycling and the lowest area fraction of <100> texture, with a texture intensity of 43.7. In an annealing temperature range of 150 °C~400 °C, the area fraction of grains with <100> orientation increased from 1.5% to 7.2%, while the intensity value of the texture decreased from 43.7 to 11.1 with the increase of temperature.
2. The size of the sample declined with the increase of the number of cycles during the thermal cycling. The residual plastic strain of samples annealed at 150 °C, 350 °C and 400 °C were −1.6 × 10^−5^, −2.2 × 10^−5^ and −4.1 × 10^−5^ after twelve thermal cycles with ΔT = 120 °C, respectively. The dimensional stability of pure aluminum was progressively deteriorated with the increment of <100> texture and the decrement of <111> texture.
3. Thermal cycling contributed to the formation of a large amount of equiaxed grains in the microstructure of pure aluminum, which were aligned along the <100> orientation. The area fractions of the grains with <100> orientation of the samples annealed at 150 °C, 350 °C and 400 °C after thermal cycling were 18.7%, 23.7% and 30.9%, respectively. The recrystallized grains with <100> orientation promoted the formation of new equiaxed grains with <100> orientation, which could be found through microstructure analysis.
4. The residual plastic strain during thermal cycling was attributed to the orientation of grains being rotated from parallel to <111> orientation to parallel to <100> orientation. According to microstructure analysis and experimental results, dimensional stability during thermal cycling is associated with the change of the orientation of grains and the proportion of the texture with <100> orientation.

## Figures and Tables

**Table 1:** Detailed parameters of the common texture components in the aluminum alloy.

Texture	{φ_1_, φ, φ_2_}	Miller Index	Texture	{φ_1_, φ, φ_2_}	Miller Index
Cube	{0°, 0°, 0°}	{001} <100>	R–Cube_RD_	{0°, 20°, 0°}	{013} <100>
R-Cube	{45°, 0°, 0°}	{001} <110>	P	{70°, 45°, 0°}	{011} <1−22>
G/B	{20°, 45°, 0°}	{011} <4−11>	G/B_T_	{50°, 45°, 0°}	{011} <1−11>
Goss	{0°, 45°, 0°}	{110} <001>	R–Goss	{90°, 45°, 0°}	{011} <0−11>
S	{59°, 37°, 43°}	{123} <63−4>	Goss_T_	{90°, 25°, 45°}	{113} <332>
Copper	{90°, 35°, 45°}	{112} <11−1>	R–Copper	{0°, 35°, 45°}	{112} <1−10>
E	{0°, 55°, 45°}	{111} <1−10>	F	{90°, 55°, 45°}	{111} <−1−12>
Brass	{35°, 45°, 0°}	{011} <2−11>	m–Brass	{50°, 65°, 63°}	{211} <0−11>

## Data Availability

The data presented in this study are available on request from the corresponding author.
